# Caspase-8 deficiency in mouse embryos triggers chronic RIPK1-dependent activation of inflammatory genes, independently of RIPK3

**DOI:** 10.1038/s41418-018-0104-9

**Published:** 2018-04-17

**Authors:** Tae-Bong Kang, Ju-Seong Jeong, Seung-Hoon Yang, Andrew Kovalenko, David Wallach

**Affiliations:** 1 Department of Biotechnology, College of Biomedical and Health Science, Konkuk University, Chung-Ju, 27478 Korea; 2 Department of Biomolecular Sciences, The Weizmann Institute of Science, 76100 Rehovot, Israel; 3 Present Address: Systems Biotechnology Research Center, Korea Institute of Science and Technology (KIST), Gangneung, 25451 Korea

## Abstract

Deletion of the *Casp8* gene in epithelial tissues of mice results in severe inflammatory pathologies. Its ubiquitous deletion, or its specific deletion in endothelial cells, results in intrauterine death associated with capillary damage. These pathologies are all preventable by co-deletion of *Casp8* and the genes encoding either the RIPK1 or the RIPK3 protein kinase. Since activation of RIPK3 in Caspase-8-deficient cells can trigger necroptotic cell death, and since RIPK1 can activate RIPK3, it is widely assumed that the inflammatory states resulting from Caspase-8 deficiency occur as a consequence of RIPK3-induced necroptosis. Here, we report that although on a *Ripk3*-null background C*asp8* deletion in mice does not result in outright pathological changes, it triggers enhanced expression of a variety of inflammatory genes in utero, which gradually subsides after birth. Deletion of *Ripk1*, or even of only one of its two alleles, obliterates this activation. Resembling the embryonic pathology observed in RIPK3-expressing cells, the activation of inflammatory genes observed on a *Ripk3*-null background seems to be initiated in endothelial cells. Analysis of endothelial cells isolated from livers of Caspase-8-deficient embryos revealed neither an increase in the amount of RIPK1 in these cells after *Casp8* deletion, nor triggering of RIPK1 phosphorylation. These findings indicate that the triggering of inflammation by *Casp8* deletion in mice occurs, in part, independently of necroptosis or other functions of RIPK3, and rather reflects enhanced RIPK1-dependent signaling for activation of inflammatory genes.

## Introduction

Caspase-8 is unique among members of the caspase cysteine protease family with regard to the far-reaching functional consequences of its deficiency. While its activation triggers apoptotic cell death through the extrinsic cell-death pathway [1, 2], deletion of the *Casp8* gene, or that of FADD—the adapter protein to which Caspase-8 binds—results in circulatory failure and death of mice at mid-gestation, associated with damage to capillaries [3–6]. The same lethal effect in utero is observed when the *Casp8* gene is specifically deleted in endothelial cells [7]. On the other hand, its deletion in epithelial tissues such as the epidermis or the intestinal epithelium triggers a severe chronic inflammatory state post-partum, associated with massive tissue damage [8, 9].

The finding that certain pathogens have evolved mechanisms to block the function of caspases, including that of Caspase-8, has drawn considerable attention to mechanisms accounting for the inflammatory states dictated by *Casp8* deletion, and the possible real-life corollaries of these experimental pathologies [10]. Assessment of the consequences of Caspase-8 deficiency in cultured cells revealed that these cells, while resistant to apoptotic-death induction by receptors of the TNF family, display dramatically enhanced vulnerability to the induction of necroptotic death [11–13]. Since necrotic cell death yields the release of pro-inflammatory cellular components (danger-associated molecular patterns—DAMPs), it is widely assumed that the acute inflammatory pathologies observed when *Casp8* is deleted in epithelial tissues, as well as the fatal outcome of its ubiquitous deletion, result from the triggering of necrotic cell death [14]. Supporting this notion was the finding that deletion of the genes encoding either the RIPK1 or the RIPK3 protein kinase, previously shown to participate in signaling for necroptotic death, or of the pseudokinase MLKL which, once phosphorylated by RIPK3, mediates the cellular membrane rupture that triggers this death, attenuates the pathological states inflicted by Caspase-8, or FADD deficiency [13, 15–19].

Here, we analyze the impacts of *Casp8*, *Ripk1*, and *Ripk3* deletion on the intrauterine expression of inflammatory genes in mice. We show that although the outright pathological changes known to result from *Casp8* deletion depend on the function of RIPK3 [3–9], the expression of some inflammatory genes is enhanced by *Casp8* deletion even on a *Ripk3*-null background. This enhanced expression was found here to be strictly dependent on expression of RIPK1. These findings implicate a mechanism that is independent of RIPK3 or of death induction by it, yet depends on RIPK1, in the inflammatory processes triggered by Caspase-8 deficiency.

## Results

### Caspase-8 deficiency on a *Ripk3*-null background triggers upregulation of inflammatory genes in various embryonic tissues

**Fig. 1:**
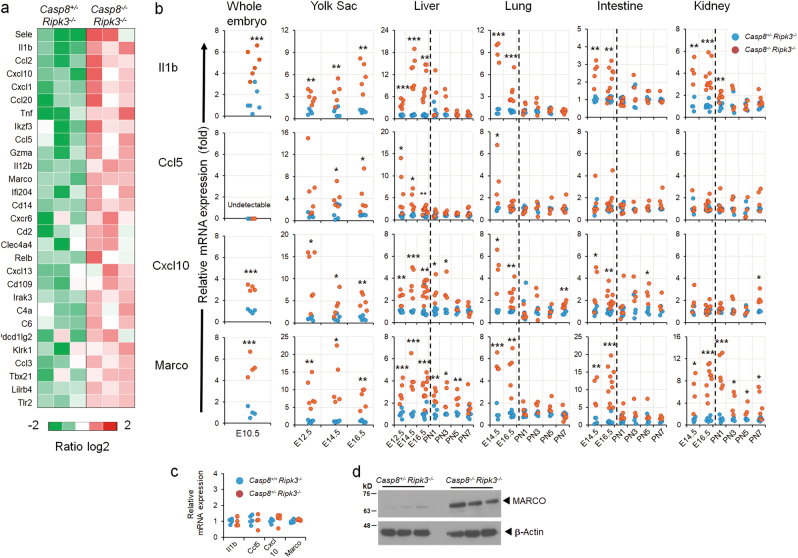
Upregulation of inflammatory genes in the embryonic tissues of *Casp8*^*−/−*^*Ripk3*^*−/−*^ mice. **a** NanoString analysis of the upregulation of immunoregulatory genes in fetal livers of *Casp8*^*−/−*^*Ripk3*^*−/−*^ mice at E16.5. Shown are the relative mRNA expression values in the livers of three *Casp8*^*−/−*^*Ripk3*^*−/−*^and three *Casp8*^*+/−*^*Ripk3*^*−/−*^ mice for genes that were upregulated by more than 2-fold (*p* < 0.05) in the *Casp8*^*−/−*^*Ripk3*^*−/−*^ samples. Red color indicates expression levels higher than the average value of the particular gene in the six examined embryonic livers. **b** Comparison of the effects of Caspase-8 deficiency on levels of the indicated mRNAs in various mouse organs at different embryonic ages (E12.5, E14.5, and E16.5) and at different times after birth (PN1, PN3, PN5, and PN7), and on their overall levels in the embryos at E10.5. Values are expression levels in the indicated organs of *Casp8*^*−/−*^*Ripk3*^*−/−*^ mice (red circles) normalized to those of *Casp8*^*+/−*^*Ripk3*^*−/−*^ mice (blue circles), assessed by analyzing at least five embryos or mice from 3 litters. ***p < 0.001, **p < 0.01, and *p < 0.05 relate to differences between the mean values for *Casp8*^*−/−*^*Ripk3*^*−/−*^ and *Casp8*^*+/−*^*Ripk3*^*−/−*^ mice. **c** Assessment of the effect of deletion of just one *Casp8* allele on the expression of inflammatory genes. Shown is expression of the indicated genes in the livers of *Casp8*^*+/+*^*Ripk3*^*−/−*^ and *Casp8*^*+/−*^*Ripk3*^*−/−*^ E16.5 mouse embryos (*n* = 4). No significant differences were found. **d** Immunoblot analysis of MARCO protein in whole liver extracts from embryos at E16.5. Data are representative of three independent experiments

Caspase-8-deficient mice on a *Ripk3*-null background (*Casp8*^*−/−*^*Ripk3*^*−/−*^) display no evident histological abnormalities in utero [13, 15] (data not shown). However, on examining the livers of these *Casp8*^*−/−*^*Ripk3*^*−/−*^ embryos at E16.5 we found, serendipitously, that their expression of the mRNA encoding the inflammatory mediator IL-1β was significantly higher than in age-matched *Casp8*^*+/−*^*Ripk3*^*−/−*^ embryonic livers. By employing the Nanostring technique to profile the genes expressed in embryonic livers of those *Casp8*^*−/−*^*Ripk3*^*−/−*^ embryos, using a panel of 547 mouse genes known to contribute to the immune response, we found that besides the increase in interleukin *Il1b*, Caspase-8 deficiency also resulted in upregulation of many other inflammatory genes (Fig. 1a).

The expression of four of these genes—*Il1b*,* Ccl5*,* Cxcl10*, and *Marco*—was then assessed in various tissues at different embryonic stages as well as post-partum. As shown in Fig. 1b, Caspase-8 deficiency resulted in enhanced expression of all four of these genes in embryonic lung, intestine and kidney, as well as in the yolk sac and was discernible, to varying extents, in these tissues as early as E12.5. Analysis of the expression of these genes in whole *Casp8*^*−/−*^*Ripk3*^*−/−*^ E10.5 embryos revealed that the expression of *Il1b*,* Cxcl10*, and *Marco* is already increased by that stage. Evidently, therefore, this increase had already occurred before or at the time of the pathological changes inflicted by deletion of *Casp8* from RIPK3-expressing embryos, which occurs at about E10.5 [3] (Fig. 1b).

After birth, the basal levels of the examined inflammatory genes were found to increase in the liver and lung, while remaining low in the intestine and kidney (Supplemental Fig. S1). In all four tissues, Caspase-8 deficiency resulted in some further increase in expression of the tested genes, for several days after birth. However, the extent of this increase was significantly lower than that observed before birth (Fig. 1b).

Deletion of just one of the Caspase-8 alleles did not result in increased expression of any of the four examined genes in the embryonic livers (Fig. 1c).

Using antibodies against MARCO for immunoblotting, we found that the amounts of MARCO protein in the livers of E16.5 *Casp8*^*−/−*^*Ripk3*^*−/−*^ embryos were much higher than in *Casp8*^*+/−*^*Ripk3*^*−/−*^ livers (Fig. 1d). Thus, upregulation of at least part of the inflammatory genes found to be activated in the *Casp8*^*−/−*^*Ripk3*^*−/−*^ mice resulted in enhanced expression of their encoded proteins.

### Inflammatory-gene activation due to Caspase-8 deficiency seems to begin preferentially in endothelial cells and then be transmitted to other cells

**Fig. 2:**
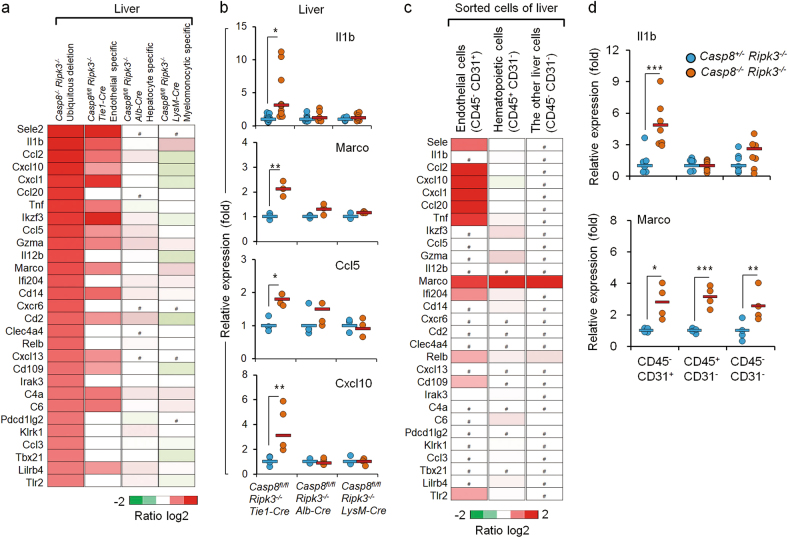
Cell-type specificity of the upregulation of inflammatory genes in Caspase-8-deficient embryonic liver. **a** NanoString analysis of the upregulation of immunoregulatory genes in embryonic livers of *Ripk3*^*−/−*^ mice at E16.5, as a result of ubiquitous deletions of the *Casp8* gene (left-most column; data are from the experiment described in Fig. 1a) or its cell-type-specific deletion by the indicated Cre transgenes. Shown are the averages of the increases in expression of the genes presented in Fig. 1a for three pairs of embryos of the following genotypes: *Casp8*^*−/−*^*Ripk3*^*−/−*^ vs. *Casp*^*+/−*^*Ripk3*^*−/−*^ from 3 litters; *Casp8*^*fl/fl*^ *Ripk3*^*−/−*^*Alb- Cre* vs. *Casp8*^*fl/fl*^ *Ripk3*^*−/−*^ (2 litters); *Casp8*^*fl/fl*^ *Ripk3*^*−/−*^*Tie1-Cre* vs. *Casp8*^*fl/fl*^ *Ripk3*^*−/−*^ (1 litter); and *Casp8*^*fl/fl*^ *Ripk3*^*−/−*^*LysM-Cre* vs. *Casp8*^*fl/fl*^ *Ripk3*^*−/−*^ (1 litter), compared to those of mouse embryos that did not express the Cre transgenes. #, undetectable. Red color indicates an expression level higher than the average value of the particular gene in the corresponding control samples. **b** Real-time PCR validation of expression levels of the indicated mRNAs in the livers of the E16.5 mice analyzed in **a**. Blue circles indicate *Casp8*^*fl/fl*^ control samples; red circles indicate tissue-specific *Caspase-8*-deficient samples. Horizontal lines indicate mean values for each group. The *Il1b* expression data were obtained by analysis of 10 pairs of *Tie1-Cre* (4 litters), 6 pairs of *Alb-Cre* (3 litters), 5 pairs of *LysM-Cre* mice (2 litters). The data about the expression of *Marco*, *Ccl5*, and *Cxcl10* were obtained by analysis of three pairs of *Tie1-Cre* (2 litters), three pairs of *Alb-Cre* (2 litters), and three pairs of *LysM-Cre* mice (1 litter). **p *<* 0.01 and *p < 0.05 relate to differences between the mean values for the *Casp8*^*fl/fl*^ *Ripk3*^*−/−*^ and *Casp8*^*fl/fl*^ *Ripk3*^*−/−*^ *Cre* mice. **c** Heat-map analysis of the relative mRNA expression, as determined by NanoString analysis, in cells sorted by FACS from three pairs (3 litters) of *Casp8*^*+/−*^ *Ripk3*^*−/−*^ and *Casp8*^*−/−*^ *Ripk3*^*−/−*^ fetal liver cells, using antibodies against the indicated cell-surface markers. Data are presented as in **a**. **d** Real-time PCR analysis of the cellular levels of mRNAs of *Il1b* and *Marco* in the indicated cell populations isolated as in **c**. Shown are the expression levels in cells derived from *Casp8*^*−/−*^ *Ripk3*^*−/−*^ (red circles) normalized to those from *Casp8*^*+/−*^ *Ripk3*^*−/−*^ mice (blue circles). Horizontal lines indicate mean values for each group. Data for *Il1b* were obtained by analysis of seven pairs (2 litters), and for *Marco* by analysis of four pairs (2 litter). ***p *<* 0.001, **p *<* 0.01, and *p < 0.05 relate to differences between the mean values for the *Casp8*^*−/−*^ *Ripk3*^*−/−*^ and *Casp8*^*+/−*^ *Ripk3*^*−/−*^ mice

To identify the liver cell type(s) in which deficiency of Caspase-8 on a *Ripk3*-null background triggers inflammatory gene expression, we used mice with a “floxed“ Caspase-8 allele (*Casp8*^*fl/fl*^) on a *Ripk3*-null background to specifically delete *Casp8* in each of the main cell types in the liver. Mating of the *Casp8*^*fl/fl*^ *Ripk3*^*−/−*^ mice with mice expressing *Cre* under control of the albumin promoter allowed deletion of *Casp8* specifically in hepatocytes. The use of mice expressing *Cre* under control of the *LysM* promoter allowed deletion of *Casp8* in the myelomonocytic cells of the liver, and the use of mice expressing *Cre* under control of the *Tie1* promoter allowed its deletion in the endothelial and hematopoietic progenitor cells. The effectiveness of *Casp8* deletion by all three *Cre* transgenes was high (Supplemental Fig. S2). Surprisingly, despite the predisposition of myelomonocytic cells to strongly express inflammatory genes, deletion of *Casp8* in these cells did not lead to upregulation of inflammatory genes in the liver. Deletion of *Casp8* in hepatocytes (the predominant liver cells) resulted in only mild activation of just two genes. In contrast, deletion of *Casp8* by expression of *Cre* under the *Tie1* promoter resulted in strong upregulation of multiple inflammatory genes, and in several of them to an extent similar to that obtained when *Casp8* was deleted ubiquitously (Fig. 2a, b).

As an alternative approach to identifying the liver cell type(s) in which Caspase-8 deficiency triggers inflammatory gene expression, we applied antibodies to CD31 (a specific cell-surface marker for endothelial cells) and to CD45 (a cell-surface marker for hematopoietic cells) in order to sort out, by FACS, distinct cell types from the livers of *Casp8*^*−/−*^*Ripk3*^*−/−*^ and *Casp8*^*+/−*^*Ripk3*^*−/−*^ E16.5 embryos. We then assessed gene expression in the isolated cells. Figure 2c shows that of the three types of cells isolated in this way, only the endothelia (CD45^−^CD31^+^ cells) expressed a variety of different inflammatory genes at very high levels. The gene for MARCO, however, was strongly upregulated in all cell types (Fig. 2c, d).

Taken together, the findings reached by both of these modes of analysis indicated that triggering of inflammatory gene expression in the liver on a *Ripk3*-null background is largely restricted to endothelial cells. Once activated, however, some of these genes are apparently able to upregulate the expression of inflammatory genes in other types of liver cells.

### Upregulation of inflammatory genes in *Casp8*^*−/−*^*Ripk3*^*−/−*^ embryos yields systemic expression of inflammatory mediators

**Fig. 3:**
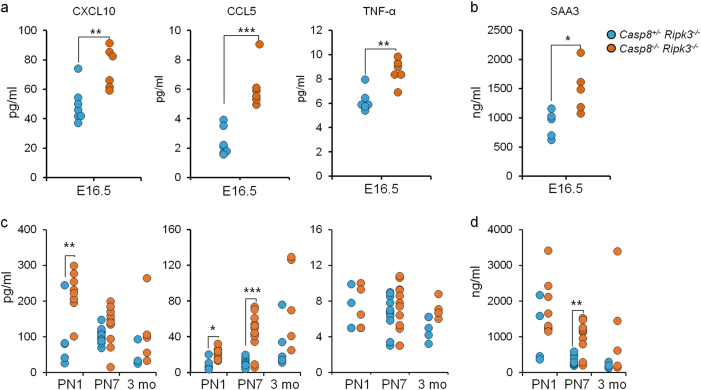
Serum expression of inflammatory mediators in *Casp8*^*−/−*^*Ripk3*^*−/−*^ mice. Serum concentrations of the cytokines CXCL10, CCL5, TNF-α, and the acute-phase protein SAA3 (**a**, **b**) in fetal blood of E16.5 embryos and in blood withdrawn postnatally at the indicated ages (**c**, **d**). Shown are values of the indicated proteins in plasma samples collected from more than five pairs (from 5 litters) of *Casp8*^*+/−*^*Ripk3*^*−/−*^ and *Casp8*^*−/−*^*Ripk3*^*−/−*^ mice, as determined (**a**, **c**) by multiplex ELISA and by conventional ELISA (**b**, **d**). ***p *<* 0.001, **p *<* 0.01, and *p < 0.05 relate to differences between the mean values for the *Casp8*^*−/−*^*Ripk3*^*−/−*^ and *Casp8*^*+/−*^*Ripk3*^*−/−*^ mice

The dissimilar findings obtained by the above two approaches used to identify the cell type in which Caspase-8 deficiency triggers activation of inflammatory genes, as well as the multiplicity of tissues in which such activation was found, suggested that the activation occurs in part in a non-cell-autonomous manner. To define the operative mechanism, we applied ELISA to determine the extent to which inflammatory mediators occur in the sera of the embryos. We found that expression levels of the chemokines CXCL10 and CCL5, the pro-inflammatory cytokine TNF and the acute-phase protein SAA3 in the sera of *Casp8*^*−/−*^*Ripk3*^*−/−*^ embryos were all increased to a significantly greater extent than in the sera of *Casp8*^*+/−*^*Ripk3*^*−/−*^ embryos (Fig. 3a, b). These results are consistent with the observed upregulation of genes encoding soluble pro-inflammatory mediators in the Caspase-8-deficient endothelium (Fig. 2), and may well account for the apparently non-cell-autonomous activation of inflammatory genes in all other cell types in the liver (Fig. 2c, d).

Some increase in CXCL10, CCL5, and SAA3 was also observed in the sera of *Casp8*^*−/−*^*Ripk3*^*−/−*^ mice during the first week after birth, but had abated by the time the mice were 3 months old (Fig. 3c, d). This increase is thus temporally distinct from the increase in inflammation and in serum cytokines in *Casp8*^*−/−*^*Ripk3*^*−/−*^ mice that is found to occur rather late after birth, as a consequence of a lymphoproliferative syndrome that they develop [13, 15, 19].

### The increased expression of inflammatory genes due to Caspase-8 deficiency is dependent on RIPK1

**Fig. 4:**
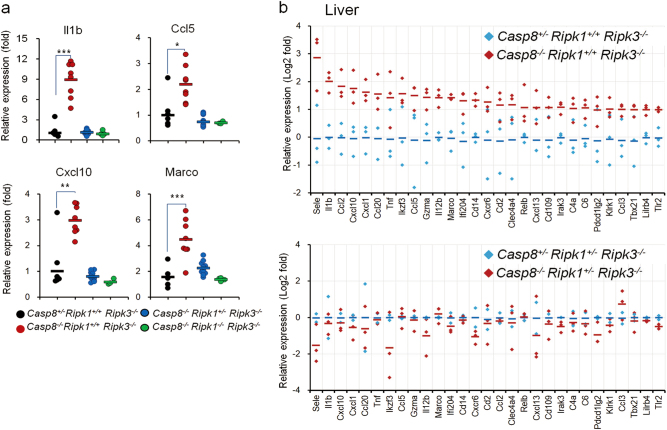
RIPK1-dependence of inflammatory gene upregulation in the embryonic *Casp8*^*−/−*^*Ripk3*^*−/−*^ liver. **a** Real-time PCR analysis of the cellular mRNA levels of the indicated genes in fetal livers of mice with the indicated genotypes at E16.5. Each circle corresponds to the average ratio of gene expression in the livers of embryos with the indicated genotypes to that of *Casp8*^*+/−*^*Ripk1*^*+/+*^ *Ripk3*^*−/−*^ embryos, obtained in four independent experiments, as follows: 5 (3 litters) *Casp8*^*+/−*^*Ripk1*^*+/+*^ *Ripk3*^*−/−*^ to 4 (3 litters) *Casp8*^*−/−*^ *Ripk1*^*+/+*^ *Ripk3*^*−/−*^ mice; 1 (1 litter) *Casp8*^*+/−*^*Ripk1*^*+/+*^ *Ripk3*^*−/−*^ and 1 (1 litter) *Casp8*^*−/−*^ *Ripk1*^*+/+*^ *Ripk3*^*−/−*^ mice and 2 (1 litter) *Casp8*^*−/−*^ *Ripk1*^*+/−*^ *Ripk3*^*−/−*^ to 1 (1 litter) *Casp8*^*−/−*^ *Ripk1*^*−/−*^ *Ripk3*^*−/−*^ mice; 3 (2 litters) *Casp8*^*−/−*^ *Ripk1*^*+/+*^ *Ripk3*^*−/−*^ mice and 5 (3 litters) *Casp8*^*−/−*^ *Ripk1*^*+/−*^ *Ripk3*^*−/−*^ mice to 1 (1 litter) *Casp8*^*−/−*^ *Ripk1*^*−/−*^ *Ripk3*^*−/−*^ mice; 4 (2 litters) *Casp8*^*−/−*^ *Ripk1*^*+/−*^ *Ripk3*^*−/−*^ mice to 2 (2 litters) *Casp8*^*−/−*^ *Ripk1*^*−/−*^ *Ripk3*^*−/−*^ mice. Horizontal lines indicate mean values for each group. ***p *<* 0.001, **p *<* 0.01, and *p < 0.05 relate to differences between the mean values for the experimental and control groups of mice. **b** Nanostring analysis of the effect of *Casp8* deletion on the expression of the genes presented in Fig. 1b in mice with two *Ripk1* alleles (upper panel: data are from the experiment described in Fig. 1b) or only one 1 *Ripk1* allele (lower panel). Data are presented as mean ± SEM of 2 (2 litters) *Casp8*^*+/−*^*Ripk1*^*+/−*^ *Ripk3*^*−/−*^ mice; 3 (3 litters) *Casp8*^*−/−*^*Ripk1*^*+/−*^ *Ripk3*^*−/−*^ mice; 3 (3 litters) *Casp8*^*+/−*^*Ripk1*^*+/+*^ *Ripk3*^*−/−*^ mice; and 3 (3 litters) *Casp8*^*−/−*^*Ripk1*^*+/+*^ *Ripk3*^*−/−*^ mice, normalized to the average levels in the corresponding *Casp8*^*+/−*^ mice

Having shown that Caspase-8 deficiency in mice dictates upregulation of some inflammatory genes independently of RIPK3, we proceeded to examine how this upregulation is affected by deletion of the gene encoding RIPK1. We found that whereas in mice expressing both alleles of *Ripk1* the deletion of *Casp8* on a *Ripk3*-null background resulted in marked upregulation of *Il1b*,* Ccl5*,* Cxcl10*, and *Marco* expression, no such increase was observed in the livers of embryos that were also deficient in RIPK1 or even in only one of the two *Ripk1* alleles (Fig. 4a). Nanostring analysis using an immunoregulatory gene panel confirmed that deletion of just one of the two *Ripk1* alleles obliterated the upregulation of the inflammatory genes induced by Caspase-8 deficiency (Fig. 4b).

### Caspase-8 deficiency does not cause increased expression of RIPK1 in endothelial cells, nor does it seem to trigger RIPK1 phosphorylation

**Fig. 5:**
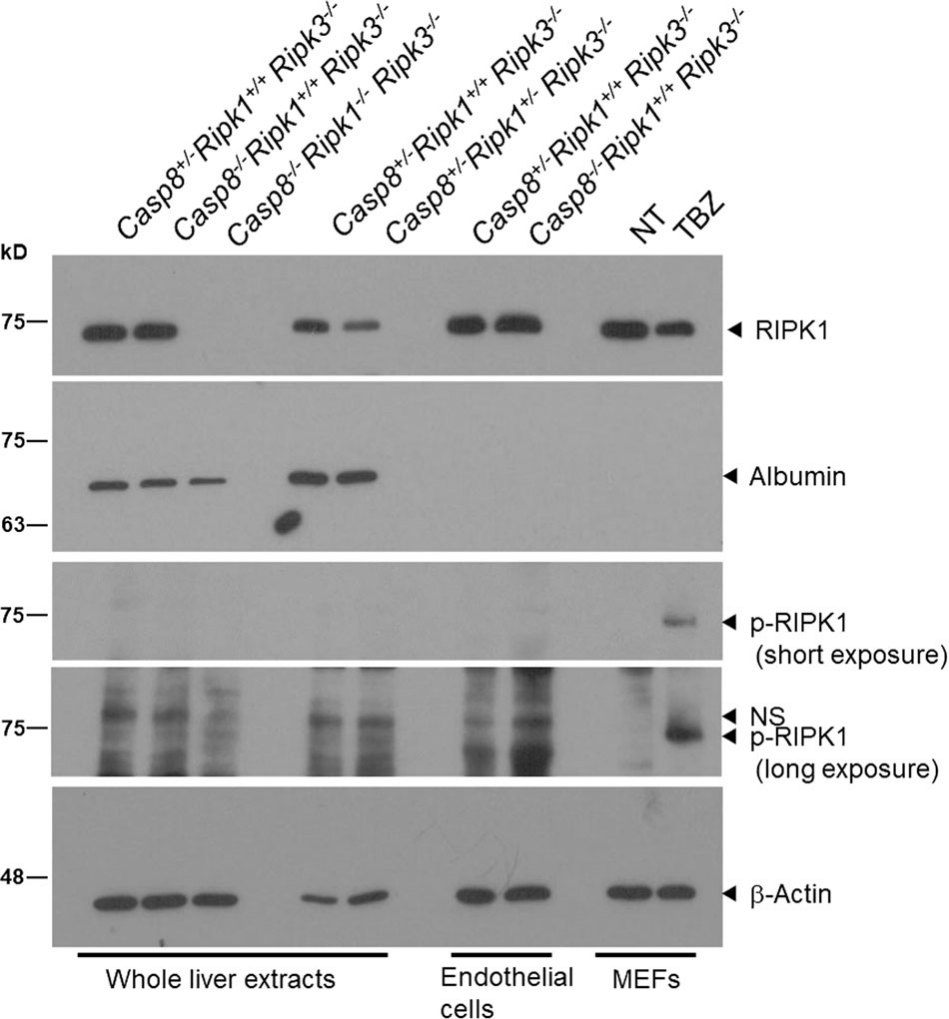
Assessment of the effects of Casp8 deletion on RIPK1 expression and phosphorylation in embryonic endothelial cells. Western blot assessments of the total amounts of RIPK1 and the amounts of RIPK1 phosphorylated at Serine 166 (p-RIPK1), in embryonic livers of mice of the indicated genotypes at E16.5 and in endothelial cells isolated from embryonic livers. Immortalized MEFs, in which RIPK1 phosphorylation was triggered by combined treatment with TNF-α, zVAD and BV6 (TBZ) as described in “Materials and methods”, served as a positive control for detection of p-RIPK1. Albumin and β-actin were blotted as loading controls of the amounts of hepatocyte extracts and for total protein, respectively. Shown are representative results of three independent experiments. NS non-specific band

Caspase-8 has been shown to respond to various stimuli by cleaving RIPK1, thereby arresting its signaling activities [20–22]. Given our finding that the upregulation of inflammatory genes in our Caspase-8-deficient embryos is strictly dependent on their adequate expression of RIPK1, it seemed possible that this upregulation reflects cessation of the constitutive downregulation of RIPK1 expression through Caspase-8-mediated RIPK1 cleavage. In such a case, RIPK1 levels in the embryonic endothelial cells, in which the upregulation of inflammatory genes occurs, would be expected to increase. To examine this possibility, we designed a two-step antibody-assisted enrichment protocol to isolate the endothelial cells from embryonic livers at E16.5. As shown in Fig. 5, western analysis using an anti-RIPK1 antibody, enabling us to reliably assess a change in RIPK1 expression resulting from deletion of one of the *Ripk1* alleles, revealed no difference between the amounts of RIPK1 in endothelial cells isolated from the livers of *Casp8*^*+/−*^*Ripk3*^*−/−*^ and of *Casp8*^*−/−*^*Ripk3*^*−/−*^ embryos.

To this western analysis we then further applied an antibody that specifically recognizes RIPK1 molecules phosphorylated at Serine 166. This antibody allowed easy detection of phosphorylated RIPK1 found in extracts of wild-type mouse fibroblasts that were stimulated by TNF in the presence of a caspase inhibitor and a cIAP antagonist. It did not, however, interact with comparable amounts of RIPK1 found in the *Casp8*^*−/−*^*Ripk3*^*−/−*^ embryonic endothelial cells (Fig. 5).

## Discussion

The prevailing conception of the various pathological changes resulting from Caspase-8 deficiency is that they are all mediated through a common mechanism in which sequential activation of RIPK1 and RIPK3 results in phosphorylation of the Mixed Lineage Kinase Domain-Like (MLKL) pseudokinase. The latter mediates necroptotic death of Caspase-8-deficient cells, and then DAMPs released by the dying cells trigger the inflammatory changes associated with these various pathologies. Our present findings imply that the inflammatory processes inflicted by Caspase-8 deficiency also involve activation of inflammatory genes by a mechanism that does not depend on the function of RIPK3 or on the death induction by it, but is nevertheless still dependent on RIPK1.

These finding are consistent with the large amount of evidence for contributions of RIPK1 in a variety of ways to the initiation of several signaling pathways that lead to activation of inflammatory genes, including the canonical NF-κB pathway and MAP-kinase cascades. Such participation occurs both in a way that depends on the RIPK1 protein-kinase function and independently of it [23–26]. Our findings are also consistent with several studies in which Caspase-8 deficiency was found to facilitate such pro-inflammatory functions of RIPK1 without leading to cell death [27–30].

As expected from the fact that Capase-8 deficiency in RIPK3-deficient embryos does not result in outright pathological changes, the extent of upregulation of inflammatory genes observed in these embryos was far lower than that observed after *Casp8* deletion in mice that do express this protein kinase. The marked increase in inflammation seen in the RIPK3-expressing mice might result in part from the initiation of necroptosis. However, in view of growing evidence that RIPK3 can also facilitate inflammation in ways that do not depend on cell death [26, 30–33], as well as some evidence for death-independent pro-inflammatory functions of the RIPK3 target protein MLKL [34–36], it seems likely that part of this enhanced inflammation does not result from necroptotic death but rather reflects non-deadly pro-inflammatory functions of RIPK3 and MLKL.

Our findings suggest that both the increase in expression of inflammatory proteins in RIPK3-deficient mouse embryos and the fatal outcome of Caspase-8 deficiency in embryos that do express RIPK3 are initiated in the same type of cell, namely, the endothelial cells. That finding, together with the similar timing of those changes, raises the possibility that despite marked differences in their consequences, the two mechanisms are interlinked. There are several possible links between the two. One intriguing possibility is that the RIPK3-independent activation of genes in response to deficiency of Caspase-8 is a precondition for those RIPK3-dependent pathologies that develop spontaneously in response to Caspase-8 deficiency. A plausible example of such interdependence is provided by our finding that TNF is one of the genes upregulated by Caspase-8 deficiency on a *Ripk3*-null background. TNF expression is a precondition for triggering of the RIPK3-dependent damage in E10.5 mouse embryos [37]. It is thus reasonable to hypothesize that the RIPK3-independent generation of TNF in embryos in response to Caspase-8 deficiency is the trigger for the RIPK3-dependent effects. The spontaneous development of pathological RIPK3-dependent consequences of Caspase-8 deficiency in adult mice may likewise depend on prior RIPK3-independent upregulation of some inflammatory genes.

An alternative possible cause of the apparent temporal and spatial co-occurrence of RIPK3-dependent and RIPK3-independent consequences of Caspase-8 deficiency in embryos is the existence of a mechanistic step that is common to both sets of consequences. This possibility raises the need to reconsider the identity of the Caspase-8 molecular target that accounts for the pro-inflammatory effect of deficiency in this caspase. In prior discussions, it was suggested that this consequence of Caspase-8 deficiency reflects the arrest of Caspase-8-mediated cleavage of one or more of three signaling proteins: RIPK1, RIPK3, and the deubiquitinating enzyme CYLD [13, 21, 38]. Our finding that Caspase-8 deficiency results in activation of pro-inflammatory genes even in the absence of RIPK3 clearly rules out an exclusive role for arrest of RIPK3-cleavage in this process. The contribution of CYLD to signaling mediated by RIPK1 + RIPK3 occurs through strengthened association of these two proteins and has to do with enhancement of their kinase function [39]. Our observation that inflammatory proteins became activated in the absence of RIPK3 and apparently without phosphorylation of RIPK1 casts doubt on the possibility that CYLD cleavage is a player in the regulation of this process.

Finally, our finding that endothelial cells isolated from Caspase-8-deficient mice and from mice not lacking Caspase-8 contain equal amounts of RIPK1 casts doubt on the arrest of Caspase-8-mediated RIPK1 cleavage as a possible player in this eventuality. In fact, we cannot exclude the possibility that the observed upregulation of inflammatory genes as a result of Caspase-8 deficiency does not reflect the prevention of cleavage of any substrate of this protease, but rather reflects the arrest of some non-enzymatic function of Caspase-8. It has indeed been shown that Caspase-8, besides acting as a protease, also contributes to signaling by serving as a scaffold for the assembly of other signaling proteins [36, 40–42].

The cellular expression of RIPK3 is subject to modulation by inducing agents and is increased in various inflammatory diseases [43–45]. Our finding that the initiation of inflammation in vivo as a consequence of Caspase-8 deficiency also occurs in the absence of RIPK3, albeit to a much lower degree than in the presence of RIPK3, implies that our cells possess the ability to respond in mild or alternatively in intense manner to pathogens that block caspase action. Such a graded response would offer more discriminatory assistance in overcoming infection. Restraining the expression of RIPK3, or of proteins acting downstream of it, can allow Caspase-8-deficient cells to initiate a defensive response through inflammatory changes not associated with their own damage. Should more powerful means of defense become necessary, upregulation of the expression of RIPK3 and of proteins downstream of it would allow these defense mechanisms to be supplemented by others that do lead to self-destruction of cells and tissues.

## Materials and methods

### Mice

All mice used in this study were on a C57BL/6 background. Mouse strains carrying a knocked-out *Casp8* allele (*Casp8*^*−/+*^) [3] and a conditional *Casp8* allele (*Casp8* ^*fl/+*^) [7] were established in our laboratory. The use of mice expressing Cre under control of the albumin promoter (*Alb-Cre*) [46], the lysozyme M gene promoter (*LysM-Cre*) [47], and the Tie1 promoter (*Tie1*^*−*^*Cre*) [48] for deletion of the *Casp8* gene specifically in hepatocytes, in liver myelomonocytic cells, and in both endothelial cells and hematopoietic progenitor cells, respectively, has been described [7]. Mice deficient in *Ripk1* were obtained from Dr. Michelle Kelliher [49] and mice deficient in *Ripk3* from Dr. Vishva Dixit [50]. Embryos and yolk sacs were isolated on the indicated days of timed pregnancies and genotyped by PCR using tail genomic DNA, as described [7]. All animal experiments were approved by the Institutional Animal Care and Use Committees at The Weizmann Institute of Science.

### FACS sorting

Fetal livers were dissected and incubated in RPMI medium containing collagenase D (50 U/ml; Thermo Fisher Scientific) and 5% fetal bovine serum for 40 min at 37 °C. Collagenase-treated tissues were dissociated into single cells by pipetting and filtering through a 70-μm cell strainer (BD Biosciences). Red blood cells were removed by treatment with a buffered ammonium chloride solution and then with mouse BD Fc Block^TM^ (BD Biosciences) for 5 min.

For the experiment presented in Fig. 2c and d, the cells were stained with allophycocyanin (APC)-conjugated anti-mCD45 antibody (17-0451, BD Biosciences) and fluorescein isothiocyanate (FITC)-conjugated anti-mCD31 antibody (11-0311, BD Biosciences), allowing sorting for endothelial cells (CD45^−^CD31^+^), hematopoietic cells (CD45^+^CD31^−^), and other liver cells (CD45^*−*^CD31^*−*^).

The effectiveness of *Casp8* deletion in individual cell types (Supplemental Fig. S2) was assessed by isolation of endothelial cells by FACS sorting after staining as above, and isolation of macrophages after staining with APC-conjugated anti-F4/80 antibody (17-4801, Thermo Fisher Scientific) and with phycoerythrin (PE)-conjugated anti-CD45 antibody (12-0415, Thermo Fisher Scientific).

For the experiment presented in Fig. 5, endothelial cells from the dissociated fetal liver tissue were isolated using a MACS Cell Separation LS column (Miltenyi Biotech), using anti-CD31 antibody (BD Pharmingen) and anti-rat IgG MicroBeads (Miltenyi Biotech). They were then further enriched by FACS sorting using anti-CD31 antibody as described above. After sorting, purity testing confirmed that more than 96% of the isolated cells were endothelial.

In all cases, staining with antibodies was carried out for 20 min on ice. The cells were then washed with cold FACS buffer (2% FBS, 0.01% sodium azide) and resuspended in the same buffer for cell sorting using a FACSAria II (BD Biosciences). Immediately before FACS analysis, the DNA dye 7-amino-actinomycin D was added for staining of dead cells. The isolated cells were harvested by centrifugation at 300 × *g* for 5 min at 4 °C and were either frozen immediately and kept at −80 °C pending extraction of total RNA or genomic DNA, or extracted with detergent for western blot analysis.

### Assessing the extent of deletion of the floxed *Casp8* allele

The extent of deletion of floxed *Casp8* alleles in tissues and cells isolated from mice that express *Cre* under control of various promoters was assessed by real-time PCR, using DNA extracted with a QIAamp DNA Micro Kit (Qiagen), according to the manufacturer’s protocol. The assay was performed in a total reaction volume of 20 μl containing 30 ng DNA, 300 nM oligonucleotide primers, 100 nM oligonucleotide 3′-Minor groove binder (MGB) probes, and 10 μl of TaqMan Universal PCR Master Mix (Applied Biosystems). The PCR procedure was done by subjecting the reaction mixture to incubation for 10 min at 95 °C, and then to 40 cycles of 15 s at 95 °C and of 1 min at 60 °C on an ABI Prism 7300 System (Applied Biosystems). Primers and probes were designed using Primer Express software (Applied Biosystems). The oligonucleotide primers and probes used to hybridize the floxed region in the *Casp8* gene were 5′-TCCTGTGCTTGGACTACATCC-3′ (sense), 5′-TTCCCGCAGCCTCAGAAATAG-3′ (antisense), and 5′-6-FAM- AGAAGCAGGAGACCATCGAGGATGC-MGB-3′ (probe). Data were normalized on the basis of quantification of the non-loxP-flanked region (Exon8) in the *Casp8* gene using 5′- GCGCAGACCACAAGAACAAAG-3′ (sense), 5′-CCTTCCCATCCGTTCCATAGAC-3′ (antisense), and 5′-6-FAM-TGCTTCATCTGCTGTATCCTATCCCA-MGB-3′ (probe). The comparative threshold cycle method (ΔCT) was used to quantify the deletion of *Casp8*.

### RNA extraction from tissues or sorted cells

Embryos were dissected on the indicated embryonic day and their organs were isolated and placed in RNAlater Stabilization Solution (Ambion) until processed for RNA extraction. RNA was extracted using the RNeasy Mini kit (Qiagen), according to the manufacturer’s instructions. RNA from FACS-sorted cells was extracted using the RNeasy Micro Kit (Qiagen). The quality of the RNA was assessed on the Agilent 2100 Bioanalyzer (Agilent Technologies).

### Real-time RT-PCR

Total RNA from tissue or cells was reverse-transcribed using the SuperScript II First-Strand Synthesis System (Invitrogen). Real-time PCR was performed using the following TaqMan primers and probe sets (Applied Biosystems).

**Table:** 

Target gene	Catalog number
*Il1b*	Mm00434228_m1
*Cxcl10*	Mm00445235_m1
*Ccl5*	Mm01302428_m1
*Marco*	Mm00440265_m1

RNA expression was normalized to the housekeeping gene *Hprt1* and calculated using the ddCt algorithm.

### Profiling of gene expression

NanoString technology with nCounter Digital Analyzer and nSolver software (NanoString Technologies) was used to assess gene expression in the indicated cell types and tissue. Briefly, RNAs were hybridized with the nCounter Mouse Immunology Panel (GXA-MIM1-12) and data were analyzed according to the recommendations of NanoString Technology. Total RNA (100 ng) isolated from fetal liver and from FACS-sorted hematopoietic cells (CD45^+^ CD31^−^), or total cell lysates of 6000 sorted endothelial cells (CD45^−^ CD31^+^) and of the other fetal liver cells (CD45^−^ CD31^−^) in 1.5 μl of buffer RLT (Qiagen), were hybridized with capture and reporter probes.

### Chemokine and cytokine assays in the blood of embryonic and adult mice

Blood samples were collected from the umbilical cords of mouse embryos and from the orbital sinuses of adult mice were collected into EDTA-coated tubes and spun at 1500 × *g* for 15 min at 4 °C. Plasma concentrations of CXCL10, CCL5, and TNF-α were measured using the Magnetic Luminex Screening Assay system (R&D Systems) and a MAGPIX system (Luminex). Plasma concentrations of serum amyloid A3 were determined by ELISA (Merck Millipore), according to the manufacturer’s instructions.

### Stimulation of RIPK1 phosphorylation in mouse embryonic fibroblasts (MEFs)

MEFs immortalized by expression of the SV40 large T antigen were cultured in Dulbecco’s Modified Eagle’s Medium supplemented with 10% FBS, 100 U/ml penicillin, and 100 mg/ml streptomycin. RIPK1 phosphorylation was stimulated in the MEFs by their treatment for 3 h with TNF (1000 U/ml), together with the bivalent IAP (inhibitor of apoptosis protein) antagonist BV6 [51] and the caspase inhibitor z-VAD-fmk (both from WuXi AppTec) at concentrations of 1 and 20 μM, respectively.

### Immunoblotting

For the experiment presented in Fig. 1d, whole liver extracts were prepared by boiling fetal liver in 1% SDS-lysis buffer (50 mM Tris, pH 8, 150 mM NaCl, 1% SDS) followed by ultrasonication for 10 s. For the experiment shown in Fig. 5, whole liver extracts, extracts of FACS-purified endothelial cells, and extracts of MEFs were prepared in RIPA buffer (50 mM Tris-HCl (pH 7.4), 150 mM NaCl, 1% TritonX-100, 0.5% sodium deoxycholate and 0.1% SDS) containing a cocktail of protease inhibitors (Sigma). Protein concentration in the extracts was determined using the BCA Protein Assay Kit (Thermo Fisher Scientific). Proteins were then separated by SDS-PAGE and transferred onto a nitrocellulose membrane. The protein-loaded membrane was blocked with 10% milk in PBS containing 0.05% Tween 20, and incubated overnight with the primary antibody and then for 1 h with HRP-conjugated secondary antibody (Jackson ImmunoResearch). Binding of the latter was detected using an enhanced chemiluminescence kit (Thermo Fisher Scientific). The following primary antibodies were used: anti-MARCO (Santa Cruz Biotechnology, sc-68623), anti-phospho-RIPK1 (Serine 166; rodent specific) (Cell Signaling Biotech, 31122), anti-RIPK1 (BD Bioscience, 610459), anti-albumin (Cell Signaling Biotech, 4929), and anti-β-actin (Sigma, A5541).

### Statistical analysis

Statistical analysis was performed with GraphPad Prism 5.0 (GraphPad Software) Differences between two groups were evaluated by two-tailed unpaired Student’s *t*-test and differences among more than two groups were evaluated by one-way ANOVA, followed by the Tukey’s post hoc test. Values of *p* < 0.05 were considered statistically significant.

## Electronic supplementary material

Legends of Supplemental Figures(DOCX 31 kb)

Supplemental Figure S1(JPG 184 kb)

Supplemental Figure S2(JPG 106 kb)
