# Supplementary material for: Caspase-8 deficiency in mouse embryos triggers chronic RIPK1-dependent activation of inflammatory genes, independently of RIPK3
Source: Cell Death Differ. 2018 Apr 17;25(6):1107–17. doi: 10.1038/s41418-018-0104-9 (PMC5988659; doi:10.1038/s41418-018-0104-9)
Supplement: Supplementary file 1 — Legends of Supplemental Figures(DOCX 31 kb) [file 41418_2018_104_MOESM1_ESM.docx]

**Legends of Supplemental Figures**

**Supplemental Figure S1 Gene expression changes in various organs during development of *Casp8^+/-^ Ripk3^-/-^* mice**

Levels of *Il1b, Ccl5, Cxcl10* and *Marco* mRNAs were determined by real-time RT-PCR. Data are presented as fold change normalized to the earliest examined time point (E12.5 for liver and yolk sac, E14.5 for the other organs). At least 4 mice from more than 2 litters were analyzed. Each symbol represents one mouse.

**Supplemental Figure S2 Effectiveness of *Casp8* deletion by the various tissue-specific *Cre* transgenes**

Effectiveness of deletion of the floxed *Casp8* gene in whole livers of postnatal day 1 mice expressing *Cre* under the hepatocyte-specific albumin promoter (*Alb-Cre*), and in macrophages and endothelial cells isolated by FACS from livers of postnatal day 1 mice expressing *Cre* under the myelomoncytic specific lysozyme M promoter (*LysM-Cre*), and under the promoter of *Tie1*, which is specifically expressed in endothelial cells and hematopoietic progenitors (*Tie1-Cre*), respectively, was assessed as described in Materials and Methods. Values are means ± SD of % deletion in 3 newborn mice from each genotype. Given the fact that hepatocytes constitute about 70% of the cells in mature mice, and less in newborn mice, the extent of deletion observed in whole livers of the *Alb-Cre* expressing mice implies that deletion in the hepatocytes of these mice was within the range of effectiveness observed for deletion dictated by *LysM-Cre* in the macrophages.
