# Supplementary figures and images for: Caspase-8 deficiency in mouse embryos triggers chronic RIPK1-dependent activation of inflammatory genes, independently of RIPK3
Source: Cell Death Differ. 2018 Apr 17;25(6):1107–17. doi: 10.1038/s41418-018-0104-9 (PMC5988659; doi:10.1038/s41418-018-0104-9)

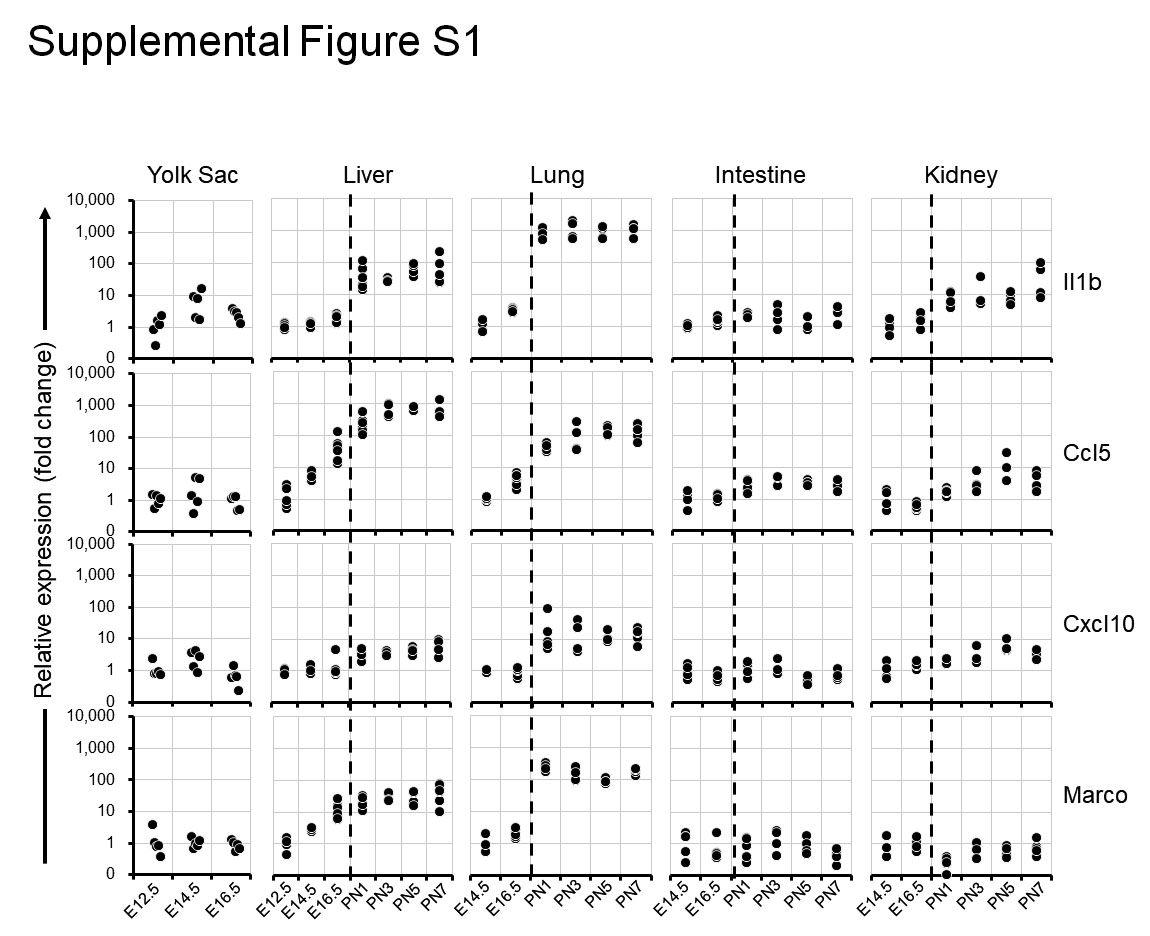

Supplement: Supplementary file 2 — Supplemental Figure S1(JPG 184 kb) [file 41418_2018_104_MOESM2_ESM.jpg]

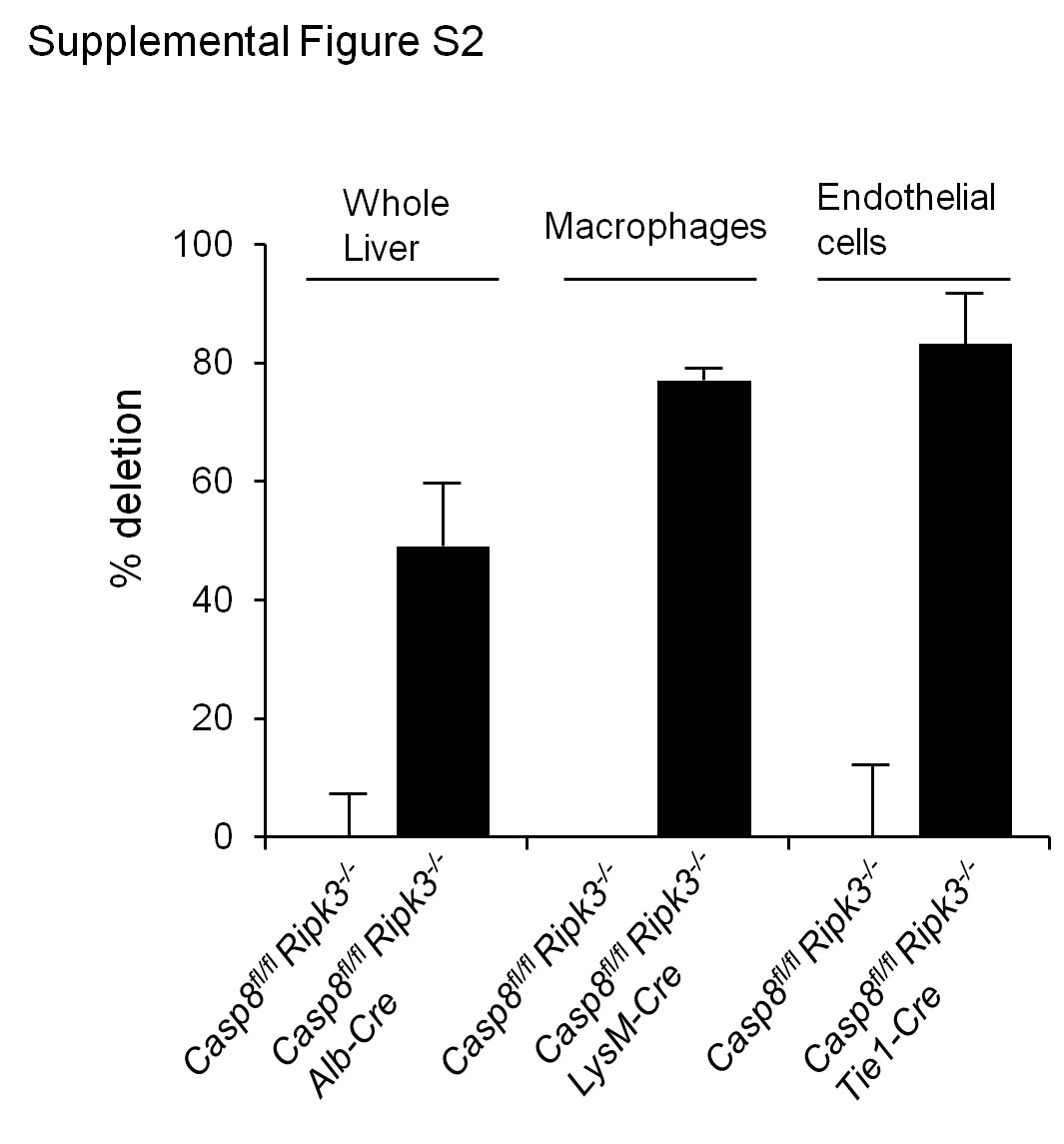

Supplement: Supplementary file 3 — Supplemental Figure S2(JPG 106 kb) [file 41418_2018_104_MOESM3_ESM.jpg]
